# Exopolysaccharides From *Streptococcus thermophilus* ST538 Modulate the Antiviral Innate Immune Response in Porcine Intestinal Epitheliocytes

**DOI:** 10.3389/fmicb.2020.00894

**Published:** 2020-05-19

**Authors:** Hiroya Mizuno, Kae Tomotsune, Md. Aminul Islam, Ryutaro Funabashi, Leonardo Albarracin, Wakako Ikeda-Ohtsubo, Hisashi Aso, Hideki Takahashi, Katsunori Kimura, Julio Villena, Yasuko Sasaki, Haruki Kitazawa

**Affiliations:** ^1^Food and Feed Immunology Group, Laboratory of Animal Products Chemistry, Graduate School of Agricultural Science, Tohoku University, Sendai, Japan; ^2^Livestock Immunology Unit, International Education and Research Center for Food and Agricultural Immunology (CFAI), Graduate School of Agricultural Science, Tohoku University, Sendai, Japan; ^3^Department of Medicine, Faculty of Veterinary Science, Bangladesh Agricultural University, Mymensingh, Bangladesh; ^4^Laboratory of Immunobiotechnology, Reference Centre for Lactobacilli (CERELA-CONICET), San Miguel de Tucumán, Argentina; ^5^Scientific Computing Laboratory, Computer Science Department, Faculty of Exact Sciences and Technology, National University of Tucuman, San Miguel de Tucumán, Argentina; ^6^Laboratory of Animal Health Science, Graduate School of Agricultural Science, Tohoku University, Sendai, Japan; ^7^Laboratory of Plant Pathology, Graduate School of Agricultural Science, Tohoku University, Sendai, Japan; ^8^Plant Immunology Unit, International Education and Research Center for Food and Agricultural Immunology (CFAI), Graduate School of Agricultural Science, Tohoku University, Sendai, Japan; ^9^Food Microbiology and Function Research Laboratories, Meiji Co., Ltd., Kanagawa, Japan; ^10^Laboratory of Fermented Foods, Graduate School of Agriculture, Meiji University, Kanagawa, Japan

**Keywords:** *Streptococcus thermophilus* ST538, exopolysaccharides, *epsB*, *epsC*, gene-knockout, PIE cells, antiviral immunity

## Abstract

It was reported that exopolysaccharides (EPSs) from lactobacilli are able to differentially modulate mucosal antiviral immunity. Although research has described the ability of EPSs derived from *Streptococcus thermophilus* to modulate the mucosal immune system, their impact on antiviral immunity was less explored. In this work, we investigated the capacity of the EPS-producing *S. thermophilus* ST538 to modulate the innate antiviral immune response triggered by the activation of the Toll-like receptor 3 (TLR3) in porcine intestinal epitheliocytes (PIE cells). Moreover, in order to study the immunomodulatory potential of *S. thermophilus* ST538 EPS, we successfully developed two mutant strains through the knockout of the *epsB* or *epsC* genes. High-performance liquid chromatography and scanning electron microscopy studies demonstrated that the wild type (WT) strain produced as high as 595 μg/ml of EPS in the skim milk medium, while none of the mutant strains (*S. thermophilus* Δ*epsB* and Δ*epsC*) were able to produce EPS. Studies in PIE cells demonstrated that the EPS of *S. thermophilus* ST538 is able to significantly improve the expression of interferon β (*IFN-β*), interleukin 6 (*IL-6*), and C-X-C motif chemokine 10 (*CXCL10*) in response to TLR3 stimulation. The role of EPS in the modulation of antiviral immune response in PIE cells was confirmed by comparative studies of cell free culture supernatants and fermented skim milks obtained from *S. thermophilus* Δ*epsB* and Δ*epsC*. These results suggest that *S. thermophilus* ST538 could be used as an immunobiotic strain for the development of new immunologically functional foods, which might contribute to improve resistance against viral infections.

## Introduction

*Streptococcus thermophilus* is a Gram-positive, non-pathogenic lactic acid bacterium, commonly used as starter culture for yogurt manufacturing. Several strains of *S. thermophilus* have been shown to produce exopolysaccharides (EPS). Since it is naturally produced by a microorganism with the “generally regarded as safe” status, EPS produced by *S. thermophilus* is considered to be safe and therefore, it has been widely used to improve rheological and sensory attributes of fermented food products (Petersen et al., 2000; Fialho et al., 2008; Iyer et al., 2010; Caggianiello et al., 2016). The EPS production among the majority of *S. thermophilus* strains varies from 20 to 600 μg/ml in milk-based medium under optimum conditions (Petersen et al., 2000; Vaningelgem et al., 2004). Therefore, the dairy industry has been interested in the search and study of strains capable of the greatest production of EPS.

Structurally, the EPS produced by *S. thermophilus* are heteropolysaccharides made up of oligosaccharide repeating unit synthetized in the cytoplasm by the action of a number of glycosyltransferases and branched chains containing glucose and galactose, rhamnose and sometimes N-acetyl-glucosamine and fucose (Doco et al., 1990; Petit et al., 1991; Dan et al., 2009). Genome-scale studies revealed that the EPS biosynthetic pathway in *S. thermophilus* involves the sugar uptake system, nucleotide sugar synthesis, polysaccharide synthesis, and export of EPS, which are controlled by several housekeeping genes and a cluster of EPS-related genes (Laws et al., 2001; Wu et al., 2014; Cui et al., 2017; Alexandraki et al., 2019; Xiong et al., 2019). In lactic acid bacteria (LAB), the typical EPS gene cluster was described to contain five highly conserved genes *epsA*, *epsB*, *epsC*, *epsD*, and *epsE*, and a variable region, which includes the genes for polymerases, flippases, and a variable number of glycosyltransferases and other modifying enzymes [reviewed in Zeidan et al. (2017)]. It has been shown that the phosphoregulatory system, constituted by *epsB*, *epsC*, and *epsD*, is responsible for the control of the polysaccharide assembly machinery (Yother, 2011; Grangeasse, 2016). Recent genomic studies have demonstrated a similar organization in the EPS clusters of *S. thermophilus* (Alexandraki et al., 2019). Although the genome analysis of several *S. thermophilus* strains revealed variable sizes in the EPS gene clusters, the 5′ and the 3′ ends were highly conserved and their differences are located mainly in the middle of the clusters. Moreover, the *epsA*, *epsB*, *epsC*, and *epsD* genes were found at the 5′ end in all the EPS gene clusters studied (Alexandraki et al., 2019).

In the last decades, EPS produced by beneficial bacteria have received attention because of their contribution to health maintenance (Laiño et al., 2016). Some recent studies demonstrated that EPS produced by probiotic bacteria are able to modulate the systemic and mucosal immune responses, and in turn to provide direct health-promoting benefits. In this regard, EPS-producing strains such as *S. thermophilus* ST1342, ST1275, and ST285 have shown to exert immunomodulatory effects on acute ulcerative colitis and to have the ability to improve the intestinal barrier function restricting adhesion and invasion of pathogens (Purwandari and Vasiljevic, 2009; Kebouchi et al., 2016). It was demonstrated that the purified EPS produced by *S. thermophilus* MN-BM-A01 protected the intestinal barrier integrity after the challenge with LPS and significantly reduced the expression of pro-inflammatory cytokines in a murine model of colitis induced by dextran sulfate sodium (DSS) (Chen et al., 2019). Marcial et al. (2017) reported that *S. thermophilus* CRL1190 and its EPS are able to adhere to the stomach gastric mucosa, reduce the adhesion of the pathogen *Helicobacter pylori* and significantly diminish the expression of pro-inflammatory factors. Although research has described the ability of EPSs derived from *S. thermophilus* to modulate the mucosal immune system in the contexts of inflammation and bacterial infections, their impact on antiviral immunity was less explored.

Taking into consideration that the EPS produced by lactobacilli have been shown to beneficially modulate the systemic and mucosal antiviral responses and to reduce the severity of viral infections (Kitazawa et al., 1998; Makino et al., 2006, 2010, 2016; Nagai et al., 2011; Kanmani et al., 2018a, b), the aim of this work was to evaluate whether the EPS-producing *S. thermophilus* ST538 or its EPS were able to modulate the innate antiviral immune response triggered by the activation of the Toll-like receptor 3 (TLR3) in porcine intestinal epitheliocytes (PIE cells). Moreover, in order to conclusively demonstrate the role of the EPS in the immunomodulatory effect of the ST538 EPS strain, two mutant strains for the *epsB* or *epsC* genes were successfully developed.

## Materials and Methods

### Bacteria Strain and Growth Conditions

*Streptococcus thermophilus* ST538 was obtained from the Food Research and Development Center, Meiji Dairies Corporation (Tokyo, Japan). This strain has been used for the commercial production of yogurt because of its great capacity of acidification when compared to other strains of the same species. *S. thermophilus* ST538 was cultured at 37°C for 16 h in M17 broth medium (Becton, Dickinson and Co., Franklin Lakes, New Zealand) supplemented with 1% of lactose (LM17 medium) or skim milk medium.

The LM17 broth medium was used for genetic manipulation and bacterial stimulation experiments. The stock bacteria cultures were inoculated at 1% (vol/vol) in fresh LM17 medium at 37°C for 16 h. These cultures were also used for inoculation of LM17 medium.

The skim milk medium was used for EPS extraction and quantification. *S. thermophilus* was cultured in skim milk medium supplemented with 2% (vol/vol) casamino acid (Nippon Shinyaku Ltd., Kyoto, Japan) or 0.1% peptide from casein hydrolysates (CE90GMM, Nippon Shinyaku Ltd., Kyoto, Japan). In the case of skim milk medium with casamino acid, the stock bacteria cultures were inoculated at 1% (vol/vol) into skim milk medium that was prepared with 10% (vol/vol) of skim milk powder and 0.05% (vol/vol) yeast extract (Oxoid Limited, United Kingdom) in water and sterilized at 121°C for 7 min. The cultures were maintained at 37°C for 16 h. These cultures were used for inoculation of the skim milk medium supplemented with 2% (vol/vol) casamino acid at 43°C. The preparation and sterilization of skim milk medium was performed according to the method described by Kitazawa et al. (1998). *S. thermophilus* was also cultured in the skim milk medium with 0.1% peptide from casein hydrolysates that was sterilized at 95°C for 2 min for EPS quantification. The preparation and sterilization of skim milk medium was performed according to the method described by Yamauchi et al. (2019).

### Sequence Analysis of epsAD Fragment of *S. thermophilus* ST538

**FIGURE 1:**
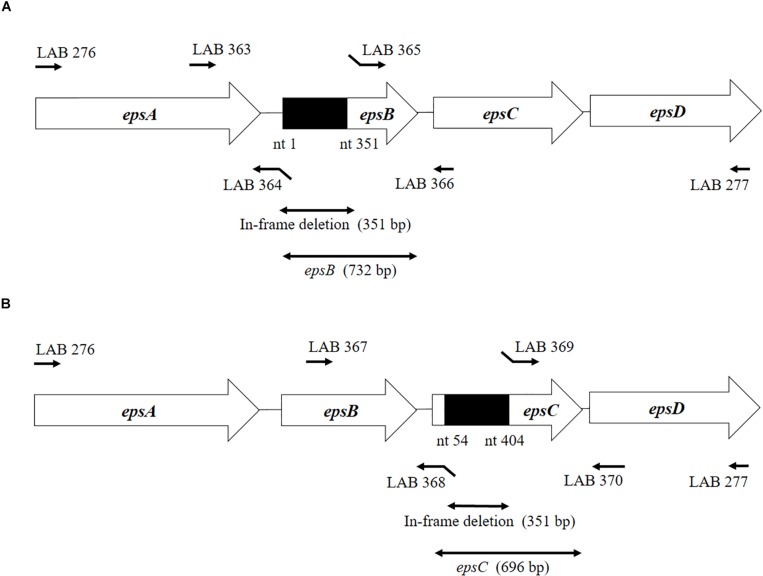
Development of non-producing exopolysaccharide (eps) mutant strains from *Streptococcus thermophilus* ST538. **(A)** Summary of the strategy used to the development of *S. thermophilus* Δ*epsB* by double-crossover. **(B)** Summary of the strategy used to the development of *S. thermophilus* Δ*epsC* by double-crossover. Primer sequences are listed in Table 1.

According to Kyoto Encyclopedia of Genes and Genomes (KEGG), the sequences of the *epsA-B-C-D* genes of the EPS locus are highly conserved among *S. thermophilus* strains. The DNA fragment corresponding to this region, called *epsAD* fragment, was amplified and sequenced with primers LAB276 and LAB277 (Table 1 and Figure 1). The DNA sequences were analyzed by Genetic Analyzer (3130, Applied Biosystems). The sequences were submitted to GeneBank under the accession numbers LC529489, LC529490, LC529491, and LC529492.

**TABLE 1:** The list of the primers used for mutants development.

Primer	Sequence
LAB276	GAAATGAGCATCGTGGTTCC
LAB277	CGATGTGCTCTTGAATGACC
LAB278	CTACTGGGTTTGCAGAGTTG
LAB279	GCACGAATTAACTTCACATC
LAB280	CAACAGAGCTAATCGCAATC
LAB281	GTCCTATTCCACCTAATCCAAC
LAB284	GGAAAGTGCCTCTCAAGCTATC
LAB285	CGTTGTGACATCTTCAACTTTTGTCACCTTC
LAB286	CGCGTATCCGCATCAATCAGAAG
LAB293	GCGTATGCACAACACTCAATTTGCTTTG
LAB294	CCTAAGAAACTGAAAGCAGCGAAA
LAB295	GGTTCATATGGTTGCAAGCGA
LAB296	CCAAAGAGCTTTGGTTTGAGGACA
LAB298	GGGCGTAACTTTCACCAATGAGG
LAB318	GCCAGAGCATTAATGGTATCTAGAGACA
LAB321	GTGTTGAAATCGACATACTAGCATTGCTAC
LAB322	CCCTTGTGTGAAAGCTTGATCATTATGGACT
LAB323	CGCTCGAAGAAGCTAAATTACCAGAGTCA
LAB324	GCTAGCATAAGAGCCACTCAAGACCATAG
LAB334	GCTCGTGAAGGAAAATCAACGACA

### Development of *S. thermophilus* ST538 epsB and epsC Knockout Mutants

The construction of the *epsB-* and *epsC-*knockout mutants (*S. thermophilus*Δ*epsB* and *S. thermophilus*Δ*epsC*, respectively) from wild type *S. thermophilus* ST538 (*S. thermophilus* WT) was performed according to the method described by Yamauchi et al. (2019). Briefly, the 351-bp in-frame deletion of *epsB* was obtained by PCR using primers LAB363 and LAB364, and then the primers LAB365 and LAB366 (Table 1 and Figure 1). Those fragments were combined by overlap extension PCR using primers LAB363 and LAB366 (Table 1 and Figure 1). This combined fragment was digested by *Bam*HI and *Xho*I, and inserted to a thermo-sensitive replicon containing plasmid pG^+^host6 (Appligene, Pleasanton, CA, United States) (Table 2) using the DNA Ligation Kit Ver1 (Takara Bio Inc., Tokyo, Japan) to generate the pG^+^epsB plasmid. This vector construction was performed in *Escherichia coli* TG1. The pG^+^epsB plasmid was first electroporated into *Lactococcus lactis* IL1403 followed by electroporation into *S. thermophilus* ST538. Similarly, the 351-bp in-frame deletion of *epsC* was obtained using primers LAB367, LAB368, LAB369, and LAB370 (Table 1 and Figure 1), and pG^+^epsC was electroporated into *S. thermophilus* ST538 following same protocol described for pG^+^epsB. Finally, the double crossover event was performed according to the method described by Biswas et al. (1993). The deletion 351-bp from the genome of *S. thermophilus*Δ*epsB* and Δ*epsC* knockout mutants were confirmed by sequence analysis.

**TABLE 2:** Bacterial strains and plasmids used.

		Phenotypic characters	References
Strain	*Streptococcus thermophilus* ST538	EPS producing strain	This study
Plasmids	pG^+^host6	Thermos-sensitive replicon^1^	Maguin et al., 1996
	pG^+^epsB	*epsB* deleted fragment of pG^+^host6	This study
	pG^+^epsC	*epsC* deleted fragment of pG^+^host6	This study

*^1^Erthromycin-resistant.*

### Scanning Electron Microscope (SEM)

Scanning Electron Microscope (SEM) was used to analyze the bacterial surfaces of wild type *S. thermophilus* ST538 (WT) and *S. thermophilus*Δ*epsB* and Δ*epsC* knockout mutants. For this purpose, the bacteria were cultured for 16 h at 37°C in LM17 broth, then centrifuged (6,000 × *g*, for 5 min at 5°C), and the pellets were diluted 10-fold with PBS. Samples were dropped into Membrane Filter polycarbonate of 0.2 μm × 13 mm (ADVANTEC^®^), and the bacterial cells were placed on the filter using suction filtration. These filters were allowed to stand in 2% (vol/vol) glutaraldehyde for 1 h at room temperature to fix the cells. The membrane was immersed in sterile water to remove excess glutaraldehyde. Thereafter, the membrane was soaked serially in 50, 60, 70, 80, 85, 90, 99, and 100% (anhydrous) ethanol for 20 min for each case. Finally, the membrane was immersed in T-butanol followed by lyophilization, platinum-palladium vapor deposition, and image capturing with a SEM (Hitachi SU8000, Tokyo, Japan) at 3 kV.

### Exopolysaccharide Concentration

The quantification of EPS production was performed according to the method described by Makino et al. (2006). *S. thermophilus* WT, Δ*epsB* and Δ*epsC* strains cultures were inoculated (2%) into the skim milk medium supplemented with 0.1% peptide from casein hydrolysates and maintained at 43°C for 24 h. The samples of skim milk medium were collected at every 2 h. The acidity of culture medium was measured according to the method described by Yamauchi et al. (2019). For EPS determination, 10 g skim milk medium were mixed with trichloroacetic acid (10% vol/vol) (FUJIFILM Wako Pure Chemical Co., Japan), left for 10 min at 4°C and then centrifuged (16,000 × *g*, 20 min, 4°C). Supernatants were collected into 2 ml eppendorf tube, mixed with 1.5 times volume of cold Ethanol (99.5%, FUJIFILM Wako Pure Chemical Co., Japan), left for 2 h at 4°C and then centrifuged (16,000 × *g*, 20 min, 4°C). The pellets were resuspended with MilliQ water and purified using a Millex-HV PVDF 0.45 mm filter (Merck Co., Tokyo, Japan). These suspensions were used to measure EPS amount using standard curve by high performance liquid chromatography (HPLC) on OHpak SB-806HQ (Showa Denko K.K., Japan) and SB-G (Showa Denko K. K., Japan). The HPLC system used Aquity H-class (Waters Co., Milford, MA, United States) and the detector RI 2414 (Waters Co., Milford, MA, United States). The elution was performed with distilled water containing 0.2 M of NaCl at 40°C at a flow rate of 0.5 ml/min.

### Extraction of Crude EPS From *S. thermophilus* ST538

The extraction of crude EPS from *S. thermophilus* ST538 was performed using the LM17 medium and the skim milk medium supplemented with 2% casamino acid. For LM17 medium, the cultures of WT and Δ*epsB* and Δ*epsC* strains were inoculated at 2% (vol/vol) to fresh medium and maintained at 37°C for 16 h. These cultures were centrifuged (6,000 × *g*, 5 min, room temperature) and the supernatants were collected, then stored at −20°C for immunoassay. Then, the supernatants were mixed with equal volume of 99.5% ethanol, and the pellets were picked up and diluted to 1/10 volume of the samples. The quantification sugars in samples were performed by Phenol sulfate method described by Kitazawa et al. (1998). For skim milk medium supplemented with 2% casamino acid, the cultures of *S. thermophilus* WT, Δ*epsB* and Δ*epsC* were inoculated at 2% (vol/vol) to the fresh medium and maintained at 43°C for 24 h. The extraction of crude EPS was performed according to the method described by Kitazawa et al. (1998). Briefly, the cultures were adjusted at pH = 4.6 and centrifuged (13,000 × *g*, 20 min, 4°C). The supernatant was neutralized and heated at 105°C, 10 min and centrifuged (13,000 × *g*, 20 min, 4°C). Cold ethanol was added to the supernatant and kept at 4°C. The samples were centrifuged (13,000 × *g*, 20 min, 4°C) and the pellets were collected, and treated with DNase and RNase (each 7 μg/ml concentration, SIGMA, St. Louis, United States) for 6 h at 37°C. Samples were treated with proteinase K (Boehringer Mannheim, Germany) with a dose of 200 μg/ml for 16 h at 37°C. The samples were heated for 10 min at 105°C, mixed with ethanol and kept at 4°C for 24 h. Finally, samples were centrifuged (13,000 × *g*, 20 min, 4°C), dialyzed against water for 48 h and subjected to lyophilization.

#### PIE Cells

The PIE cell line was originally derived from intestinal epithelia isolated from an unsuckled neonatal swine (Moue et al., 2008). PIE cells are intestinal non-transformed cultured cells that assume a monolayer with a cobblestone and epithelial-like morphology and with close contact between cells during culture (Moue et al., 2008). PIE cells were maintained in Dulbecco’s modified Eagle’s medium (DMEM) (Invitrogen Corporation, Carlsbad, CA, United States) supplemented with 10% fetal calf serum (FCS), 100 U/ml streptomycin, and 100 mg/ml penicillin at 37°C in an atmosphere of 5% CO_2_ (Moue et al., 2008; Albarracin et al., 2017).

#### Immunomodulatory Effect of Streptococci in PIE Cells

The study of the immunomodulatory capacity of *S. thermophilus* WT, Δ*epsB* and Δ*epsC* strains, their cell-free culture supernatants or the EPS from *S. thermophilus* ST538 was performed in PIE cells as described previously (Albarracin et al., 2017; Kanmani et al., 2018a, b). PIE cells were seeded at 3 × 10^4^ cells per well in 12-well type I collagen-coated plates (Sumitomo Bakelite Co., Tokyo, Japan) and cultured for 3 days. After changing medium, streptococci (5 × 10^8^ cells/ml) were added and 48 h later, each well was washed vigorously with medium at least three times to eliminate all stimulants. Then cells were stimulated with poly(I:C) (60 ug/ml) for 12 h for RT-PCR studies. Similarly, PIE cells were stimulated with cell-free culture supernatants or 20, 50, or 100 μg/ml of purified EPS and then challenged with poly(I:C) (60 ug/ml) for 12 h for RT-PCR studies.

#### Quantitative Expression Analysis by Two-Step Real-Time Quantitative PCR

Two-step real-time quantitative PCR (qPCR) was performed to characterize the expression of selected genes in PIE cells as described previously (Albarracin et al., 2017; Kanmani et al., 2018a, b). TRIzol reagent (Invitrogen) was used for total RNA isolation from each PIE cell sample and, Quantitect reverse transcription (RT) kit (Qiagen, Tokyo, Japan) was used for the synthesis of all cDNAs according to the manufacturer’s recommendations. Real-time quantitative PCR was carried out using a 7300 real-time PCR system (Applied Biosystems, Warrington, United Kingdom) and the Platinum SYBR green qPCR SuperMix uracil-DNA glycosylase (UDG) with 6-carboxyl-X-rhodamine (ROX) (Invitrogen). The primers used in this study were described before (Albarracin et al., 2017; Kanmani et al., 2018a, b). The PCR cycling conditions were 2 min at 50°C, followed by 2 min at 95°C, and then 40 cycles of 15 s at 95°C, 30 s at 60°C, and 30 s at 72°C. The reaction mixtures contained 5 μl of sample cDNA and 15 μl of master mix, which included the sense and antisense primers. According to the minimum information for publication of quantitative real-time PCR experiments guidelines, β-actin was used as a housekeeping gene because of its high stability across porcine various tissues (Nygard et al., 2007; Bustin et al., 2009). Expression of β-actin was used to normalize cDNA levels for differences in total cDNA levels in the samples.

#### Statistical Analysis

The qRT-PCR raw data were log-transformed followed by normality check by Kolmogorov-Smirnov test and convergence by clubs rejection test. One-way ANOVA was performed in GraphPad prism v5.1 followed by calculating the Fisher’s least significant difference for multiple mean comparisons were defined as significant at *p* < 0.05.

## Results

### Development of Δ*epsB* and Δ*epsC* Mutants From *S. thermophilus* ST538

**FIGURE 2:**
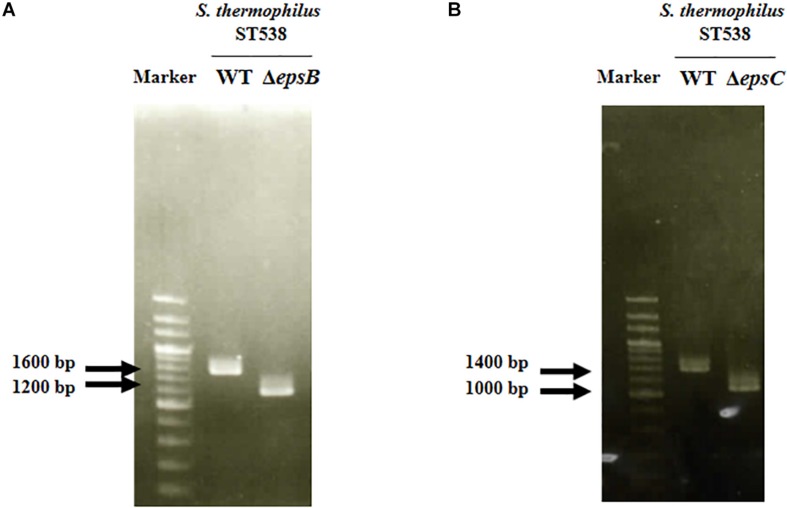
Development of non-producing exopolysaccharide (eps) mutant strains from *Streptococcus thermophilus* ST538. **(A)** Confirmation of the deletion of 351-bp in the *epsB* gene by PCR analysis. **(B)** Confirmation of the deletion of 351-bp in the *epsB* gene by PCR analysis. Wild type (WT) and Δ*epsB* and Δ*epsC* mutants from *S. thermophilus* ST538 were compared.

We analyzed the sequence *epsA-D* in *S. thermophilus* ST538 by using the Applied Biosystems 3130 Genetic Analyzer and the result showed that there was high base sequence homology with *epsA*, *epsB*, *epsC*, and *epsD* from *S. thermophilus* CNRZ1066; being 98, 96, 92, 97% respectively (data not shown). Then, it was postulated that EpsB, EpsC, and EpsD constituted a phosphorylation-dependent regulatory system of EPS production in the ST538 strain. *S. thermophilus*Δ*epsB* (Figure 1A) and Δ*epsC* (Figure 1B) were produced by double-crossover and the deletion of 351-bp in the *epsB* and *epsC* genes, respectively. In order to confirm the targeted deletion of nucleotides in *S. thermophilus*Δ*epsB* and Δ*epsC* strains, we performed PCR analysis (Figure 2) and sequencing (Supplementary Figure S1). As expected, the amplicon length of *epsB* in *S. thermophilus*Δ*epsB* was decreased when compared to *S. thermophilus* WT (Figure 2A). Similarly, the amplicon length of *epsC* in *S. thermophilus*Δ*epsC* was decreased when compared to the wild type strain (Figure 2B). The reduction of DNA length in approximately 400-bp for both mutant strains indicated the successful deletion of *epsB* and *epsC* genes. The DNA sequencing of both *S. thermophilus*Δ*epsB* and Δ*epsC* strains also confirmed the deletion of 351-bp in the *epsB* and *epsC* genes, respectively (Supplementary Figure S1).

**FIGURE 3:**
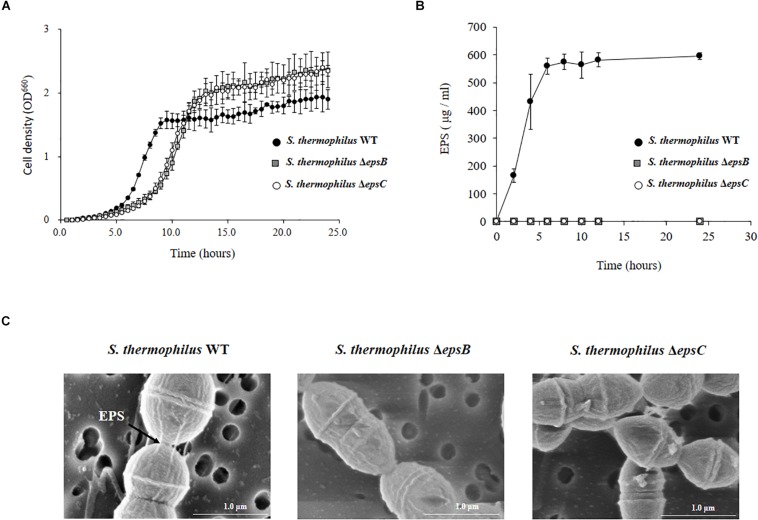
Development of non-producing exopolysaccharide (eps) mutant strains from *Streptococcus thermophilus* ST538. **(A)** Growth curve in LM17 medium, **(B)** EPS production in skim milk medium, and **(C)** electron microscope analysis of wild type (WT) and Δ*epsB* and Δ*epsC* mutants from *S. thermophilus* ST538.

We next evaluated the potential changes in the phenotype of *S. thermophilus* WT induced by the deletion of 351-bp in the *epsB* and *epsC* genes. We studied growth curves in LM17 medium to check the effect of the *eps* genes deletion. The Figure 3A shows the growth curves of WT, Δ*epsB* and Δ*epsC.* A delay of growth in the early logarithmic growth phase and a recover at the late logarithmic phase was observed for the both mutants strains when compared to the WT. In addition, we comparatively evaluated the production of EPS in *S. thermophilus* WT, Δ*epsB* and Δ*epsC* strains (Figure 3B). For the analysis of EPS production by *S. thermophilus* WT and the mutant strains, bacteria were cultured in skim milk medium (Supplementary Figure S2) and the EPS was measured at every 2 h by HPLC. As shown in Figure 3B, the wild type *S. thermophilus* ST538 was able to produce EPS from hour 2 and reaching a maximum production at hour 6. The EPS production by *S. thermophilus* WT was noticed as high as 595 μg/ml. On the contrary, neither *S. thermophilus* Δ*epsB* nor Δ*epsC* were able to produce detectable levels of EPS during all the studied period (Figure 3B). We also performed electron microscopic analysis in order to further evaluate the production of EPS (Figure 3C). The electron microscopic analysis demonstrated that *S. thermophilus* WT contains EPS molecules associated to its surface while both Δ*epsB* and Δ*epsC* mutant strains had no such fibrous structures on their surfaces (Figure 3C). Then, the inability of mutants Δ*epsB* and Δ*epsC* to synthetize EPS indicated the successful development of non-EPS producing *S. thermophilus* ST538 strains.

### Effect of *S. thermophilus* ST538 and Δ*epsB* and Δ*epsC* Mutants on the Innate Antiviral Immune Response in PIE Cells

**FIGURE 4:**
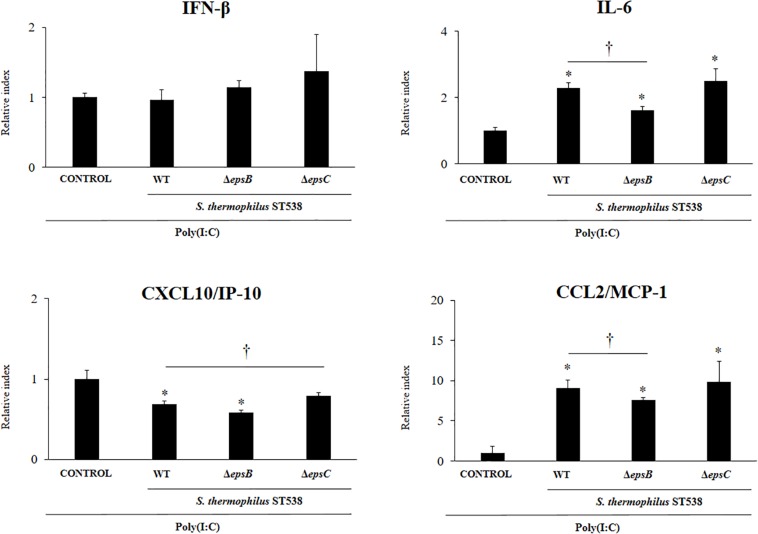
Effect of *Streptococcus thermophilus* ST538 and its exopolysaccharide (eps) mutant strains on the innate antiviral immune response triggered by Toll-like receptor 3 (TLR3) activation in porcine intestinal epithelial cells (PIE cells). Expression of *IFN-*β, *IL-6*, *CXCL-10/IP-10*, and *MCP-1/CCL2* genes in PIE cells treated with wild type (WT) *S. thermophilus* ST538 or its Δ*epsB* or Δ*epsC* mutants and challenged with the viral molecular associated pattern poly(I:C). PIE cells with no bacterial treatment and stimulated with poly(I:C) were used as controls. The results represent data from three independent experiments. Significant differences when compared to the control group ^∗^*P* < 0.05. Significant differences when compared to the indicated group ^†^*P* < 0.05.

In order to evaluate the immunomodulatory properties of *S. thermophilus* WT and the mutant strains, we performed *in vitro* experiments in PIE cells stimulated with *S. thermophilus* WT, Δ*epsB* or Δ*epsC* strains and then challenged with the TLR3 agonist and viral molecular associated pattern poly(I:C) (Albarracin et al., 2017; Kanmani et al., 2018a, b). The challenge of PIE cells with poly(I:C) significantly increased the expression of *IFN-β*, *IL-6*, *CXCL10*, and *CCL2* (Figure 4) when compared to basal levels (data not shown) as we have reported previously (Albarracin et al., 2017; Kanmani et al., 2018b). The expression of *IFN-β* in poly(I.C)-challenged PIE cells was not changed by the prestimulation with *S. thermophilus* WT or the mutants Δ*epsB* or Δ*epsC*. On the contrary, the expression levels of *IL-6* and *CCL2* were significantly increased in PIE cells treated with *S. thermophilus* WT when compared to control cells (Figure 4). The same effect was observed for *S. thermophilus*Δ*epsC* and with no significant differences when compared to the WT strain. Although *S. thermophilus*Δ*epsB*-treated PIE cells had an increased the expression of *IL-6* and *CCL2* than control cells, the values of both inflammatory factors were significantly lower than the observed for the WT strain (Figure 4). In addition, *S. thermophilus* WT was able to significantly reduce the expression of *CXCL10* in poly(I:C)-challenged PIE cells. Similarly, *S. thermophilus*Δ*epsB* reduced the expression of the pro-inflammatory chemokine while no differences were detected when *S. thermophilus*Δ*epsC*-treated and control PIE cells were compared (Figure 4).

### Effect of *S. thermophilus* ST538 EPS on the Innate Antiviral Immune Response in PIE Cells

**FIGURE 5:**
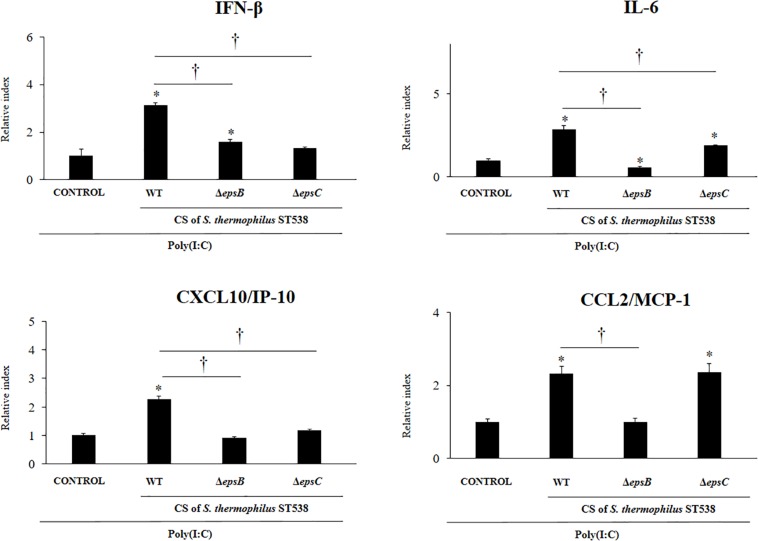
Effect of cell-free supernatants (CS) from *Streptococcus thermophilus* ST538 and its exopolysaccharide (eps) mutant strains on the innate antiviral immune response triggered by Toll-like receptor 3 (TLR3) activation in porcine intestinal epithelial cells (PIE cells). Expression of *IFN-*β, *IL-6, CXCL-10/IP-10*, and *MCP-1/CCL2* genes in PIE cells treated with CS from wild type (WT) *S. thermophilus* ST538 or its Δ*epsB* or Δ*epsC* mutants grown in LM17 medium, and challenged with the viral molecular associated pattern poly(I:C). PIE cells with no treatment and stimulated with poly(I:C) were used as controls. The results represent data from three independent experiments. Significant differences when compared to the control group ^∗^*P* < 0.05. Significant differences when compared to the indicated group: ^†^*P* < 0.05.

**FIGURE 6:**
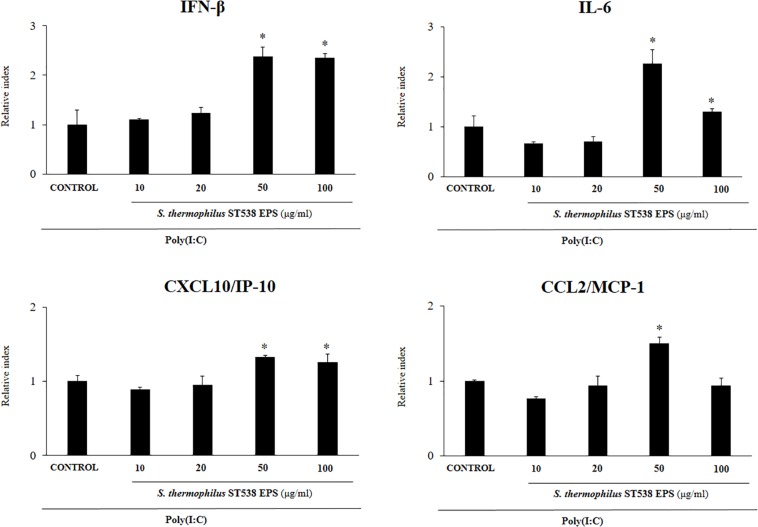
Effect of the purified exopolysaccharide (eps) from *Streptococcus thermophilus* ST538 on the innate antiviral immune response triggered by Toll-like receptor 3 (TLR3) activation in porcine intestinal epithelial cells (PIE cells). Expression of *IFN-*β, *IL-6, CXCL-10/IP-10*, and *MCP-1/CCL2* genes in PIE cells treated with different concentrations of EPS from *S. thermophilus* ST538 in LM17 medium and challenged with the viral molecular associated pattern poly(I:C). PIE cells with no EPS treatment and stimulated with poly(I:C) were used as controls. The results represent data from three independent experiments. Significant differences when compared to the control group ^∗^*P* < 0.05.

We next aimed to evaluate whether the EPS produced by *S. thermophilus* ST538 was able to modulate the innate antiviral immune response in PIE cells. For these experiments, *S. thermophilus* WT, Δ*epsB* or Δ*epsC* strains were grown in LM17 broth medium. No differences in pH values were observed when the medium inoculated with WT, Δ*epsB* or Δ*epsC* were compared (data not shown). The cell-free supernatants (CS) were used for PIE cells stimulation followed by the challenge with poly(I:C). As shown in Figure 5, the CS from *S. thermophilus* WT was able to significantly improve the expression of *IFN-β*, *IL-6*, *CXCL10*, and *CCL2* in PIE cells after the activation of TLR3. Interestingly, PIE cells treated with the CS from *S. thermophilus*Δ*epsB* had significantly lower expression levels of *IFN-β*, *IL-6*, *CXCL10*, and *CCL2* when compared to the cells stimulated with the CS from the WT strain. Moreover, *CXCL10* and *CCL2* expressions levels in the CS Δ*epsB* group were not different from control cells (Figure 5). Similarly, PIE cells treated with the CS from *S. thermophilus*Δ*epsC* had significantly lower expression levels of *IFN-β*, *IL-6*, and *CXCL10* when compared to the cells stimulated with the CS WT (Figure 5). However, no differences were found when the expression of *CCL2* was compared between CS WT and CS Δ*epsC* groups. These results indicate that the products contained in the culture supernatant of *S. thermophilus* ST538, mainly the EPS, are capable of modulating the antiviral immunity in PIE cells. Then, in order to demonstrate conclusively the immunomodulatory potential of the EPS of *S. thermophilus* ST538, we performed a second set of experiments in PIE cells by using different concentrations of purified EPS. For this purpose, PIE cells were stimulated with different concentrations of crude EPS extracted from ST538 strain grown in LM17 medium and then challenged with poly(I:C) (Figure 6). The EPS of *S. thermophilus* ST538 in concentrations superior to 50 μg/ml was able to significantly increase the expression of *IFN-β*, *IL-6*, and *CXCL10* when compared to control cells. In addition, in our experiments only the concentration of 50 μg/ml of the EPS of *S. thermophilus* ST538 enhanced the expression of *CCL2* when compared to control cells (Figure 6).

### Effect of *S. thermophilus* ST538 Fermented Skim Milk on the Innate Antiviral Immune Response in PIE Cells

**FIGURE 7:**
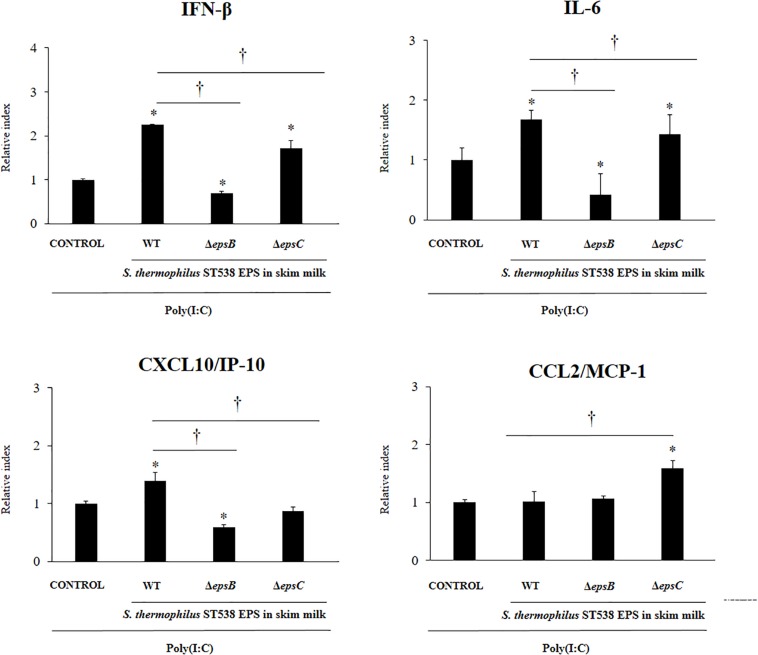
Effect of the EPS derived from skim milk fermented by *Streptococcus thermophilus* ST538 and its exopolysaccharide (eps) mutant strains on the innate antiviral immune response triggered by Toll-like receptor 3 (TLR3) activation in porcine intestinal epithelial cells (PIE cells). Expression of *IFN-*β, *IL-6*, *CXCL-10/IP-10*, and *MCP-1/CCL2* genes in PIE cells treated with the EPS from skim milk fermented by wild type (WT) *S. thermophilus* ST538 or the same fractions (EPS negative) in the cultures of Δ*epsB* or Δ*epsC* mutants, and challenged with the viral molecular associated pattern poly(I:C). PIE cells with no treatment and stimulated with poly(I:C) were used as controls. The results represent data from three independent experiments. Significant differences when compared to the control group ^∗^*P* < 0.05. Significant differences when compared to the indicated group ^†^*P* < 0.05.

Finally, we evaluated the effect of the EPS derived from a skim milk fermented by *S. thermophilus* ST538 on the antiviral immune response of PIE cells. The EPS fraction of *S. thermophilus* WT or the same fractions (EPS negative) in the cultures of the mutants *S. thermophilus*Δ*epsB* or Δ*epsC* in skim milk supplemented with 2% (vol/vol) casamino acid were used for comparisons (Figure 7). PIE cells treated with the WT EPS and then challenged with poly(I:C) had significantly higher levels of *IFN-β*, *IL-6*, and *CXCL10* when compared to control cells. No differences were found between those groups when the expression of *CCL2* was evaluated. Of note, PIE cells treated with the EPS negative fractions from Δ*epsB* or the Δ*epsC* had significantly lower expression levels of *IFN-β*, *IL-6*, and *CXCL10* when compared to the WT group (Figure 7). The most notable effect was observed for the Δ*epsB* group. No differences were found between WT EPS and EPS negative fractions from Δ*epsB* when the expression of *CCL2* was evaluated. On the contrary, EPS negative fractions from Δ*epsC* significantly increased *CCL2* expression when compared to the WT group (Figure 7).

## Discussion

Studies have demonstrated that the EPSs produced by some immunobiotic lactobacilli are able to enhance antiviral immunity (Kitazawa et al., 1998; Makino et al., 2006, 2010, 2016; Nagai et al., 2011). Moreover, those studies have reported the impact of EPSs on immune cells and had described their ability to activate macrophages, induce lymphocytes proliferation, stimulate IFN-γ production or enhance NK cell activity (Nishimura-Uemura et al., 2003; Makino et al., 2006, 2010, 2016; Nagai et al., 2011). However, the ability of EPSs derived from LAB to differentially modulate the innate antiviral immune response in IECs was not addressed in details before. In this regard, we have previously investigated the capacity of EPSs produced by the immunobiotic strains *Lactobacillus delbrueckii* OLL1073R-1 (Kanmani et al., 2018a) and *Lactobacillus delbrueckii* TUA4408L (Kanmani et al., 2018b) to beneficially modulate the innate immune response in PIE cells. This *in vitro* system has been particularly useful for the study of innate immunity in IECs, since the PIE cell line highly expresses the antiviral PRRs TLR3 and responds to poly(I:C) stimulation by producing antiviral factors and inflammatory cytokines and chemokines resembling the response to rotavirus infection (Hosoya et al., 2011; Ishizuka et al., 2016; Kanmani et al., 2018a, b). In this work, we reported and analyzed, for the first time, the capacity of the EPS-producing *S. thermophilus* ST538 strain to improve the innate antiviral immune response in IECs. Moreover, by the purification of the EPS and the successful development of Δ*epsB* and Δ*epsC* mutants derived from the ST538, we clearly demonstrated the involvement of the EPS in the immunomodulatory ability of this *S. thermophilus* strain.

Despite the vast structural diversity, bacteria produce EPS by using four different pathways [reviewed in Zeidan et al. (2017)]. One of them, the Wzy-dependent pathway, allows bacteria to produce polysaccharides by using an *en bloc* mechanism. In LAB, this is the pathway of choice for the synthesis of heteropolymeric EPS (Ryan et al., 2015; Torino et al., 2015). Genes encoding Wzy-dependent EPSs biosynthesis proteins in *S. thermophilus* are typically organized in a cluster with an operon structure and are generally chromosomal (Zeidan et al., 2017; Nourikyan et al., 2015). In the EPS cluster, the genes in the 5′ end are involved in the modulation and assembly machinery of polysaccharide biosynthesis. Those genes consist of five genes: *epsA*, *epsB*, *epsC*, *epsD*, and *epsE*, which display the highest level of overall conservation among different *Streptococcus* strains (Zeidan et al., 2017; Jolly and Stingele, 2012; Goh et al., 2011; Alexandraki et al., 2019). The *epsC* gene encodes a membrane-bound protein with a cytoplasmic C-terminal domain required for kinase activation. The *epsC* product triggers *epsD* kinase activity leading to the autophosphorylation of the tyrosine cluster and therefore, acting as a modulator component. On the other hand, the *epsB* gene encodes a phosphotyrosine protein phosphatase in *S. thermophilus*, with the ability to dephosphorylate the tyrosine cluster, acting also as a modulatory protein (Jolly and Stingele, 2012; Goh et al., 2011). Then, the current model of EPS synthesis proposes that cycling between phosphorylated and non-phosphorylated forms of the tyrosine cluster is required for proper synthesis and export of the EPS (Yother, 2011; Grangeasse, 2016; Zeidan et al., 2017). Therefore, in this work we hypothesized that the mutation of the *epsB* or *epsC* genes, would allow us to obtain two strains derived from *S. thermophilus* ST538 with the inability to produce EPS. Indeed, we successfully developed *S. thermophilus* Δ*epsB* and Δ*epsC* carrying 351-bp deletion in the *epsB* and *epsC* genes, respectively. Moreover, we demonstrated that neither of the two mutants was able to produce detectable amounts of EPS in our experimental conditions.

The lack of the ability of *S. thermophilus* Δ*epsC* to produce EPS shown here is in line with several reports studying the role of this gene in the biosynthesis of EPS in this species of bacterium. Studies evaluating the protein–protein interactions between the genetic products of *epsC*-*epsD* derived from *S. thermophilus* MR-1C demonstrated the ability of EpsD to interact with the protein tyrosine kinase EpsC (Cefalo et al., 2011a,b). In addition, the requirement of *epsC* for the phosphorylation of *epsD* was verified *S. thermophilus* CNRZ1066 (Minic et al., 2007). The work demonstrated that EPS is not synthesized in *epsC* or *epsD* mutants, indicating that both proteins are essential for EPS synthesis. Moreover, studies by Li et al. (2016), clearly demonstrated the role of the *epsC* gene on the quantity and quality of EPS production by *S. thermophilus* 05–24. The work examined the levels of *epsC* expression under different fermentation conditions and found that in the optimal fermentation condition the *epsC* gene is up-regulated in almost 3-folds.

On the other hand, the phosphotyrosine phosphatase function of purified EpsB was demonstrated in *S. thermophilus* MR-1C (Cefalo et al., 2013; Cefalo et al., 2013) and the *epsB*-*epsD* interaction was verified (Cefalo et al., 2011a,b). Recent studies evaluated the impact of fermentation conditions in the production of EPS by *S. thermophilus* ASCC1275 using transcriptomic (Wu and Shah, 2018) and proteomics (Wu et al., 2019) approaches. Those works reported that the production of EPS by the ASCC1275 strain could be improved by reducing the pH from 6.5 to 5.5. Interestingly, authors also demonstrated that there was a significant down-regulation of *epsB* at both mRNA and protein level in pH 5.5 compared to the pH 6.5 conditions. Of note, Minic et al. (2007) were able to obtain a *S. thermophilus* Δ*epsB* derived from the CNRZ1066 strain and found no differences between wild type and the Δ*epsB* mutant when the production of the EPS was compared. Moreover, the work reported an increase in the EpsE enzymatic activity in the *S. thermophilus* strain lacking the *epsB* gene. Those works indicate that the down-regulation or the elimination of the *epsB* gene in *S. thermophilus* do not modify EPS biosynthesis or can even increase it. In contrast with those results, we observed here that the *S. thermophilus* Δ*epsB* derived from the ST538 was not able to produce detectable amounts of EPS. Moreover, we recently developed another Δ*epsB* mutant strain derived from *S. thermophilus* ST499 that was not able to produce detectable amounts of EPS (unpublished results). The difference between Δ*epsB* mutants derived from CNRZ1066, ST538, and ST499 strains in their ability to produce EPS may indicate that the control mechanism of EPS production by phosphorylation/dephosphorylation may differ between strains. More detailed biochemical and genetic comparative studies using different *S. thermophilus* strains are necessary in order to conclusively clarify the molecular mechanisms involved in EPS production.

Interestingly, we also observed in this work that there was a delay of growth in the early logarithmic phase when Δ*epsB* and Δ*epsC* strains were compared to the WT *S. thermophilus.* Nourikyan et al. (2015) showed that the phosphoregulatory system CpsBCD involved in the capsule production in *S. pneumoniae* is also closely related with the cell division. Although there are marked differences between capsular and EPS production, it can be speculated that the deletion of *epsB* and *epsC* in ST538 may have affected the cell division even if, preliminary electron microscope analysis did not show significant differences in cell morphology or chain length, between wild type bacteria and mutants. Further studies are necessary to determine the exact roles of *epsB* or *epsC* genes in the cell division of *S. thermophilus* ST538.

The EPS of *S. thermophilus* ST538 was able to significantly improve the expression of *IFN-*β, *IL-6*, and *CXCL10* in PIE cells in response to TLR3 activation. On the contrary, the EPS negative fractions from skim milk or culture supernatants from the mutants *S. thermophilus* Δ*epsB* and Δ*epsC* had significantly lower levels of expression of *IFN-*β, *IL-6*, and *CXCL10* in PIE cells when compared to cells treated with the wild type EPS or supernatant. Those experiments conclusively demonstrated the role of the EPS in the modulation of TLR3-mediated immune response by the ST538 strain.

The three immune factors modulated by the EPS of *S. thermophilus* ST538, *IFN-*β, *IL-6*, and *CXCL10*, have been associated to the protection against viral infections. The activation of TLR3 in IECs triggers the IRF3 signaling conducting to the production of type I IFNs and activates NF-κB signaling that leads to up-regulation of pro-inflammatory factors. Cytokines and chemokines production induced by the TLR3-NF-κB signaling pathway in IECs include IL-6 and CXCL10, which alerts host to induce the recruitment and activation of immune cells to combat the invading pathogen (Kondo et al., 2012; Villena et al., 2016). In addition, it was reported that infection with rotavirus induces a significant up-regulation of type I IFNs by DCs and IECs (Sen et al., 2012). The binding of these IFNs to their cognate cell surface receptors activate positive feedback loops that amplifies the expression of IFNs as well as more than 300 different IFN-stimulated genes (Hoffmann et al., 2015). This IFNs release then efficiently amplifies the expression of antiviral proteins targeting a variety of viral replication steps in uninfected bystander cells. Then, considering those protective effects induced by IFNs, it can be speculated that EPS of *S. thermophilus* ST538 could have the ability to improve the resistance against enteric viruses. In support of this hypothesis, we previously demonstrated that the immunobiotic strains *Bifidobacterium infantis* MCC12 (Ishizuka et al., 2016) and *L. rhamnosus* CRL1505 (Albarracin et al., 2017) were able to significantly increase IFN-β in response to poly(I:C) challenge in PIE cells. The enhanced IFN-β induced a concomitant up-regulation of the antiviral factors NPLR3, OAS1, OASL, MX2, RNASEL, and RNASE4 in PIE cells. Moreover, the CRL1505 strain has been shown to beneficially modulate the antiviral innate immune response triggered by TLR3 activation in mice (Tada et al., 2016) and reducing the severity of viral infections in children (Villena et al., 2012). Whether *S. thermophilus* ST538 or its EPS are able to reduce the replication of enteric virus *in vitro* and *in vivo* thought the induction of IL-6, CXCL10, and type I IFNs is an open question, which we propose to address in the near future.

The global market is so far dominated by polysaccharides produced by plants and algae, however, bacteria in general and LAB in particular represent an interesting source of a polysaccharide repertoire that can be exploited for improving human and animal health. Our results suggest that EPS from ST538 strain have potential to be used for improving intestinal innate antiviral response and protecting against intestinal viruses such as rotavirus. Then, *S. thermophilus* ST538 could be used as an immunobiotic strain for the development of new immunologically functional foods, which might contribute to improve resistance against viral infections.

## Data Availability Statement

The datasets generated for this study are available on request to the corresponding authors.

## Author Contributions

YS, JV, and HK designed the study. HM, KT, RF, and MI performed the experiments. HM and LA performed the bioinformatic studies. JV, KK, HT, YS, and HK provided the financial support. JV, WI-O, HT, KK, YS, and HK contributed to data analysis and results interpretation. MI and JV wrote the manuscript. WI-O, HT, HA, KK, YS, and HK reviewed the manuscript. YS and HK approved the final version of the manuscript.

## Conflict of Interest

KK was employed by the company Meiji Co., Ltd. The remaining authors declare that the research was conducted in the absence of any commercial or financial relationships that could be construed as a potential conflict of interest.

## Supplementary Material

FIGURE S1 Development of non-producing exopolysaccharide (eps) mutant strains from *Streptococcus thermophilus* ST538. **(A)** Comparison of the *epsB* sequence in wild type (WT) and Δ*epsB* strains of *S. thermophilus*. **(B)** Comparison of the *epsC* sequence in wild type (WT) and Δ*epsC* strains of *S. thermophilus*.

FIGURE S2 Development of non-producing exopolysaccharide (eps) mutant strains from *Streptococcus thermophilus* ST538. Growth curve in skim milk medium of wild type (WT) and Δ*epsB* and Δ*epsC* mutants from *S. thermophilus* ST538.
