# Supplementary figures and images for: Exopolysaccharides From Streptococcus thermophilus ST538 Modulate the Antiviral Innate Immune Response in Porcine Intestinal Epitheliocytes
Source: Front Microbiol. 2020 May 19;11:894. doi: 10.3389/fmicb.2020.00894 (PMC7248278; doi:10.3389/fmicb.2020.00894)

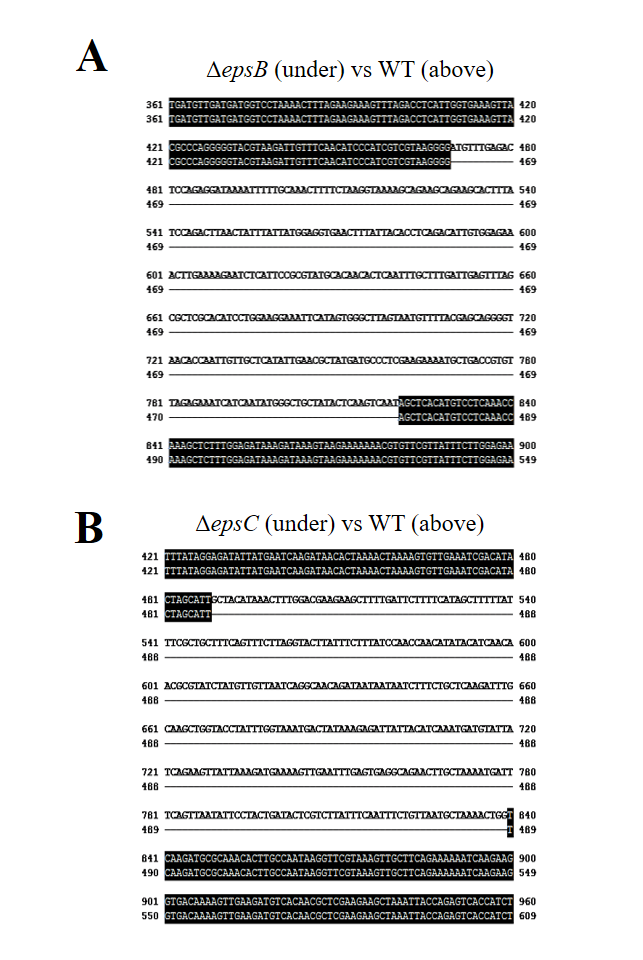

Supplement: FIGURE S1 — Development of non-producing exopolysaccharide (eps) mutant strains from Streptococcus thermophilus ST538. (A) Comparison of the epsB sequence in wild type (WT) and ΔepsB strains of S. thermophilus. (B) Comparison of the epsC sequence in wild type (WT) and ΔepsC strains of S. thermophilus. [file Image_1.TIF]

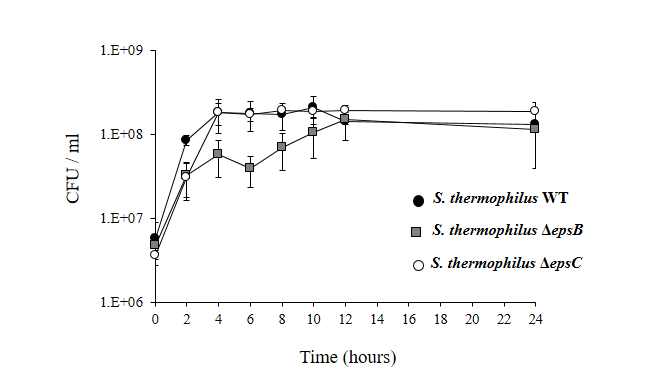

Supplement: FIGURE S2 — Development of non-producing exopolysaccharide (eps) mutant strains from Streptococcus thermophilus ST538. Growth curve in skim milk medium of wild type (WT) and ΔepsB and ΔepsC mutants from S. thermophilus ST538. [file Image_2.TIF]
